# The influence of the order and congruency of correct and erroneous worked examples on learning and (meta-)cognitive load

**DOI:** 10.3389/fpsyg.2022.1032003

**Published:** 2022-10-26

**Authors:** Lukas Wesenberg, Felix Krieglstein, Sebastian Jansen, Günter Daniel Rey, Maik Beege, Sascha Schneider

**Affiliations:** ^1^Psychology of Learning With Digital Media, Faculty of Humanities, Institute for Media Research, Chemnitz University of Technology, Chemnitz, Germany; ^2^Digital Media in Education, Institute for Psychology, University of Education, Freiburg, Germany; ^3^Educational Technology, Faculty of Arts and Social Sciences, Institute of Education, University of Zurich, Zürich, Switzerland

**Keywords:** worked example, erroneous examples, cognitive load theory, productive failure, expertise reversal effect, cascade theory, judgment of learning, belief-adjustment model

## Abstract

Several studies highlight the importance of the order of different instructional methods when designing learning environments. Correct but also erroneous worked examples are frequently used methods to foster students’ learning performance, especially in problem-solving. However, so far no study examined how the order of these example types affects learning. While the expertise reversal effect would suggest presenting correct examples first, the productive failure approach hypothesizes the reversed order to be learning-facilitating. In addition, congruency of subsequent exemplified problems was tested as a moderator of the effect of order on learning. For example, with arithmetic tasks, congruent problems target exactly the same calculation while incongruent problems refer to different calculations. Following cascade theory, a model of cognitive skill acquisition, presenting correct examples first should be more effective when the subsequent exemplified problems are different. To test the (conflicting) hypotheses, 83 university students were assigned to one of the four conditions in a 2 (correct vs. erroneous example first) × 2 (same vs. different exemplified problems) between-subject design. Learners navigated through a slideshow on the topic of Vedic mathematics consisting of explicit instruction, worked examples differing in terms of the experimental condition, and transfer problems. Although no main or interaction effects were found regarding students’ learning performance, mediational analysis offered support for the expertise reversal effect, as it indicated that there is a significant indirect effect of order *via* mental load on learning. Presenting correct examples first and erroneous examples second resulted in a lower mental load, which in turn was associated with better learning performance. In contrast, presenting erroneous examples first and correct examples second resulted in a more accurate self-assessment of learning performance. These findings offer first insights into the question of how the presentation order of different example types impacts learning and provide practical recommendations for the design of educational media. Results are discussed in light of the ongoing debate regarding the question if less guided instructional methods should precede or succeed more guided methods.

## Introduction

A learning unit that aims to teach problem-solving skills such as algebra is typically composed of multiple teaching methods that can differ regarding their level of instructional guidance (Kirschner et al., 2006). More guided methods include for example the presentation of explicit instructional text or correct worked examples, which provide structure and direct the learner in the process (Sweller et al., 1998; Loibl et al., 2017). Less guided methods, on the other hand, include for example embedding problem-solving tasks or erroneous worked examples, which have more of an explorative character and ask learners, at least partly, to develop solutions by themselves (Große and Renkl, 2007; Kapur, 2008). Various studies have already confirmed the conduciveness of each of these teaching methods for deeper learning processes (e.g., Sweller and Cooper, 1985; Kalyuga, 2007; Booth et al., 2013). In addition, research has investigated the order in which those methods should be delivered for effective learning (e.g., Van Gog et al., 2011; Kapur, 2014). The results show that order can be a decisive factor for learning success. However, no experimental study has yet investigated if and how the order of correct and erroneous worked examples affects learning-related variables such as learning success, cognitive load, and metacognitive variables such as the judgment of learning (JOL). Given the effects of order in the context of other instructional methods and findings that underline the value of using both types of examples in the educational process, this question is of great importance (e.g., Durkin and Rittle-Johnson, 2012; Heemsoth and Kleickmann, 2018). The present work aims to close this research gap and to further examine whether a potential effect of order is dependent on the congruency of the subsequent exemplified problems (i.e., whether the correct worked example represents the solution for the erroneous worked example or the solution for a different problem).

### Worked examples

Based on the cognitive load theory (CLT) by Sweller et al. (1998), the worked example effect postulates that interspersing already worked-out solutions in a learning unit instead of solely presenting problem-solving tasks can be beneficial for knowledge acquisition (Sweller and Cooper, 1985). CLT understands learning as a process in which schemata (i.e., associative knowledge structures) are either constructed by linking new information elements with prior knowledge in working memory or they get automated through repeated recall. A central assumption of CLT is that the capacity of working memory, in contrast to long-term memory, is severely limited in terms of the amount that can be processed simultaneously. Another important assumption of CLT is the distinction between different types of cognitive load, which have an additive character concerning the capacity of working memory. Recent conceptions of the CLT distinguish between the intrinsic cognitive load and the extraneous cognitive load (Sweller, 2010; Kalyuga, 2011; Kalyuga and Singh, 2016; Sweller et al., 2019; Jiang and Kalyuga, 2020). The intrinsic load is caused by the complexity of the subject at hand for the learner and by that, it is considered to be essential or productive for understanding. The complexity is in turn caused by the element interactivity (i.e., the number of information elements that have to be processed simultaneously) and the domain-specific prior knowledge. High prior knowledge lowers the number of the to-be-processed elements in working memory as the learner is familiar with the topic and recognizes the meaning of the given combination of information elements automatically. By that, several subordinate information elements are structured into a few superordinate elements which reduces the total complexity. For the design of learning materials, CLT thus suggests that the intrinsic load should be sensibly controlled and successively adapted to the learner’s expertise (Sweller et al., 1998; Kalyuga, 2007). The extraneous load, on the other hand, is not caused by the learning subject itself, but by the design of the learning environment, and by that it is considered to be unessential or unproductive for understanding the subject. For example, unimportant additions or awkward wording can increase the extraneous load. The CLT thus suggests that the extraneous load should be reduced in the design of learning materials so that the cognitive resources are not wasted (Sweller et al., 1998; Kalyuga, 2007). Therefore, the CLT generally supports guided teaching methods through which learners are directed and supported in their learning process. In contrast, strategies such as discovery learning are rejected (Kirschner et al., 2006).

Regarding worked examples, the CLT (Sweller et al., 1998) predicts a learning-promoting effect compared to problem-solving tasks since the former exerts a lower extraneous load (Sweller and Cooper, 1985). Sweller and Cooper (1985) argue that pure problem-solving is associated with a heavy load because cognitive resources are expended on solving the task at hand. The learner has to perform a means-ends analysis to find appropriate operators for transforming an actual state into the desired state. This analysis or search requires the maintenance of many elements of information, is usually very unsystematic, and proceeds slowly, in particular for novices with a low domain-specific prior knowledge (Renkl, 2014). However, according to Sweller and Cooper, the goal of the learning process is not the solution of a concrete task, but the construction of generalized problem-solving schemata by the learner, which can be applied after the learning phase. The means-ends analysis during problem-solving thus can be seen as an additional extraneous load that interferes with the learning process. In general, worked examples have the advantage of eliminating the need for a means-ends analysis during learning. The individual solution steps and operators are already given so that the learner can concentrate solely on deriving general patterns for problem-solving from the example. In this respect, worked examples are generally regarded as a comparatively guided and structure-preserving instructional method (e.g., Sweller et al., 1998; Kapur and Bielaczyc, 2012).

Many studies have empirically confirmed that a mixture of worked examples and problem-solving tasks is better than problem-solving only regarding learning performance (e.g., Sweller and Cooper, 1985; Cooper and Sweller, 1987; Paas, 1992; Carroll, 1994; Paas and Van Merrienboer, 1994; Nievelstein et al., 2013), cognitive load (e.g., Sweller and Cooper, 1985) and learning efficiency when learning performance is related to time expenditure (McLaren et al., 2008, 2016). The effect was also replicated in studies in which the control group received additional assistance (Cooper and Sweller, 1987; Schwonke et al., 2009) and in studies in which the experimental group received worked examples only instead of a mixture of worked examples and problem-solving tasks (Paas, 1992). Overall, Crissman (2006) reported a meta-analytically determined mean effect size of *d* = 0.57 of worked examples versus problem-solving tasks concerning learning performance.

### Erroneous worked examples

Traditional research on the worked example effect has mainly focused on completely correct examples (e.g., Paas, 1992; Sweller et al., 1998). Over time, however, a branch of research has emerged that investigates the learning effectiveness of erroneous worked examples (e.g., Joung et al., 2006; Große and Renkl, 2007). These can be described as worked examples that contain at least one incorrect solution step. In this respect, worked examples can be divided into correct examples (CE) and erroneous examples (EE). In the literature, various explanations are given for the learning-promoting effect of EE compared to CE (Bransford and Schwartz, 1999; VanLehn, 1999; Siegler, 2002; Joung et al., 2006; Barbieri and Booth, 2016). One widely cited explanation is offered by cascade theory (VanLehn, 1999) which describes the learning or problem-solving process as a continuous sequence of impasses, reflections, and repairments. An impasse is a kind of dead end, which means the learner finds themselves at a state during the problem-solving in which they lack the correct operators to transform the actual state into the target state. According to cascade theory and contrary to CLT (Sweller et al., 1998), such impasses are valuable for generating problem-solving schemes because they encourage reflection. Reflection expands previous knowledge. In the best case, the learner finds the correct operator and can free himself or herself from the impasse. EE may be more likely to provoke such impasse situations than CE (Große and Renkl, 2007; Heemsoth and Heinze, 2014). The assignment to comprehend why a correctly applied problem-solving operator is correct probably does not stimulate as much reflection on the valid rules and limits of this operator as the assignment to comprehend why an incorrectly applied operator is incorrect (Siegler and Chen, 2008). In the first case, it is obvious to simply rely on the given answer. In the second case, the person must delve deeper into the subject matter to generate an answer (Siegler and Chen, 2008).

Overall, empirical studies show that EE compared with CE may indeed have a positive impact on learning (Joung et al., 2006; Kopp et al., 2008; Booth et al., 2013; Heemsoth and Heinze, 2014). For example, Booth et al. (2013) showed that students presented with multiple EE on algebra developed better conceptual understanding after the intervention than those presented with multiple CE. However, some studies found either no learning advantage (Wang et al., 2015; McLaren et al., 2016; Heemsoth and Kleickmann, 2018) or, in line with the contrasting CLT framework, even a learning disadvantage compared to CE (Große, 2018). This suggests that, as with most design principles, there are factors that moderate the learning effect of EE compared to CE.

Furthermore, it was investigated how effective a mix of CE and EE is for learning. Some studies compared the effectiveness of such a condition with the exclusive presentation of EE (Booth et al., 2013; Heemsoth and Kleickmann, 2018). Heemsoth and Kleickmann (2018) found a significant difference in favor of the mixed condition, while Booth et al. (2013) found no difference. Other studies compared the effectiveness of a mixed condition with CE presentation only (Große and Renkl, 2007; Durkin and Rittle-Johnson, 2012; Booth et al., 2013; Zhao and Acosta-Tello, 2016; Huang, 2017; Heemsoth and Kleickmann, 2018; Loibl and Leuders, 2019). Here, only one of the studies found no significant differences (Huang, 2017). In the remaining studies, however, especially those which explicitly prompted the learner to compare both solution attempts, the mixed condition consistently proved to be more conducive to learning than the presentation of CE alone (Große and Renkl, 2007; Durkin and Rittle-Johnson, 2012; Booth et al., 2013; Zhao and Acosta-Tello, 2016; Heemsoth and Kleickmann, 2018; Loibl and Leuders, 2019). Overall, these studies indicate that implementing both types of examples in a learning process can be useful. However, the question remains unclear whether the order of presentation (i.e., the order of cognitive processing) is decisive for learning success and, if so, whether CE should be presented first or EE should be presented first.

### Theories on order effects

In the following, two theoretical perspectives are presented which allow deriving assumptions on the question of whether more guided instructional methods such as CE or less guided instructional methods such as EE should take place first in a learning environment.

#### Expertise reversal effect

The expertise reversal effect can be classified as a form of *aptitude-treatment interaction* (Kalyuga, 2007). This is a class of interaction effects in which a person’s dispositional characteristics influence how certain instructional interventions affect the person’s learning success (e.g., Cronbach and Snow, 1977). The expertise-reversal effect refers to the characteristics of prior knowledge and postulates that the expertise can moderate the size as well as the direction of several learning effects such as the worked example effect (Kalyuga et al., 2000; Chen et al., 2017). In its original formulation, the concept does not specify in which way expertise changes these effects as it merely states that the redundancy and thus the learning hindrance of content results from the ratio of information content to prior knowledge (Kalyuga, 2007). In general, though, studies hypothesize expertise reversal effects in such a way that guiding and structuring teaching methods such as direct instruction are predicted to be more effective for novices (Kalyuga, 2007). For experts, the same methods are predicted to be less effective or to even inhibit learning compared to reduced or challenging learning materials such as problem-solving (e.g., Kalyuga et al., 1998; Tuovinen and Sweller, 1999; Kalyuga and Sweller, 2004; Kalyuga, 2007).

That prior knowledge influences the *effect size* of certain instructional methods can be theoretically derived from CLT (Kalyuga et al., 1998; Sweller et al., 1998; Kalyuga, 2007). Accordingly, a poorly structured learning text with complex content exerts not only a high extraneous load but also a high intrinsic load on a person with little prior knowledge in the subject area leading to an overload of the working memory capacity. For example, signaling keywords can help to improve the learner’s orientation and thus at least reduce the extraneous load caused by poor structuring. This brings the learner’s overall load back into an acceptable range. If the same text is read without signaling but by a person with more prior knowledge in the subject area, the extraneous load is still high but the intrinsic load of the text is lower. The total cognitive load for the learner remains acceptable. Consequently, signaling is less important for the experienced learner.

That prior knowledge influences the *direction of effect* of certain instructional methods (i.e., it has a reversing influence) is theoretically justified in different ways depending on the given method of instruction (Kalyuga et al., 1998; Sweller et al., 1998; Renkl and Atkinson, 2003; Kalyuga, 2007). A reversing influence of prior knowledge on instructional methods that affect learner activation, just like problem-solving or EE, is explained in two ways, among others (Sweller et al., 1998; Renkl and Atkinson, 2003). On the one hand, according to Sweller et al. (1998), it can be problematic from a motivational point of view if a learning environment is based exclusively on examples. This can lead to learners processing the material only superficially, as their participation is not essential for completing the learning environment. Therefore, in later learning phases, when learners already have differentiated and largely automated solution schemes and problem-solving activities no longer provoke a large extraneous load, problem-solving tasks can be effective for learning by stimulating thinking and keeping motivation high. On the other hand, Renkl and Atkinson (2003) argue that problem-solving tasks are useful in later learning phases since the learning goal at this point is not schema construction but schema automation. This is promoted through the repeated recall of already constructed schemata. The independent solving of tasks in problem-solving likely promotes schema-recall more than worked examples. In this respect, concrete problem solving does not represent a learning-irrelevant, but rather a learning-relevant burden, not in early but later learning phases.

The expertise reversal effect was first empirically investigated and confirmed in a series of longitudinal studies by Kalyuga et al. (1998, 2000, 2001a,b). Since then, several studies have replicated the effect in the context of different methods or operationalizations of the construct of expertise and design principles (e.g., Rey and Buchwald, 2011; Nievelstein et al., 2013; for an older review see Kalyuga, 2007). Most importantly, expertise has been shown to moderate the learning facilitation of CE compared to problem-solving tasks (Tuovinen and Sweller, 1999; Kalyuga et al., 2001a,b; Kalyuga and Sweller, 2004). These studies showed that especially in early learning phases CE rather than problem-solving, and in later learning phases problem-solving rather than CE are more effective for learning.

It has also been investigated whether expertise influences the learning facilitation of EE or a mixture of EE and CE compared to CE. A few studies could not find an expertise-reversal effect (Durkin and Rittle-Johnson, 2012; Zhao and Acosta-Tello, 2016; Heemsoth and Kleickmann, 2018). However, these results might also be explained by methodological artifacts. Some studies applied median splits based on a pretest when operationalizing prior knowledge to divide the subjects into novices and experts (Zhao and Acosta-Tello, 2016; Heemsoth and Kleickmann, 2018). This is problematic because the methodology of the median split can be accompanied by a reduction in test strength (Cohen, 1983) and also because it may happen that the variance concerning prior knowledge is not particularly high in the sample (Heemsoth and Kleickmann, 2018). Some studies were able to confirm an expertise reversal effect, though (Große and Renkl, 2007; Heemsoth and Heinze, 2014; Barbieri and Booth, 2016; Beege et al., 2021). Barbieri and Booth (2016) showed that EE were only more conducive to learning for subjects with lower prior knowledge. No differences were found for subjects with higher prior knowledge. However, in this analysis, the authors did not compare the effect of EE with the effect of CE but with the overall effect of CE and problem-solving only. This makes it impossible to say whether EE is better than CE or better than problem-solving tasks for learners with low prior knowledge. Heemsoth and Heinze (2014) found an opposite pattern in their data. Learners with very low prior knowledge learned significantly better with CE. Learners with very high prior knowledge, on the contrary, learned significantly better with EE. However, it must be critically noted here that no interaction effect was tested and that only extreme groups (25%-percentile vs. 75%-percentile) were compared. A regression analysis with the condition and the continuous prior knowledge variable could not confirm any significant interaction. Große and Renkl (2007) also included prior knowledge, measured by a pretest, as a continuous variable in their regression model and did find a significant interaction effect. They found the same pattern as Heemsoth and Heinze (2014). Learners with low prior knowledge learned better with material that contained only CE. Learners with higher prior knowledge, on the other hand, learned better with material that consisted of half CE and half EE. Similarly, Beege et al. (2021) found an interaction effect in that EE improved test performance for high-prior knowledge learners, while it had no effect for low-prior-knowledge learners. In line with the expertise-reversal effect, these studies may explain the somewhat inconsistent findings on the effect of EE vs. CE by suggesting that prior knowledge is a reversing moderating factor in learning with either CE or EE.

#### Productive failure

Contrary to expertise reversal, productive failure is a learning concept developed by Kapur (2008). It classifies as a minimally-guided approach that predicts learning advantages of less structured and less directive learning environments (Kirschner et al., 2006). More specifically it relates to concepts that Loibl et al. (2017) termed PS-I approaches (problem-solving first, instruction second). These do not reject the use of directive instructions but suggest using them rather in later than earlier phases of skill and knowledge acquisition (Schwartz and Bransford, 1998; Schwartz and Martin, 2004; Kapur, 2008). As to productive failure, learners should be confronted with complex, for the learner probably unsolvable problems at the very beginning of a learning process, and in the absence of any supportive instruments or assistance. Kapur calls this first phase the exploration and generation phase since the learner should independently search for working solution strategies and apply them on a trial-and-error basis. After the learner has presumably worked through the tasks unsuccessfully, he or she is then taught the correct problem-solving strategies in a well-structured and guided learning environment. This second phase is called the direct instruction or consolidation phase (Kapur, 2010; Kapur and Bielaczyc, 2012). In this phase, further practice tasks are also presented following the instruction. The basic assumption of productive failure is thus that the problem-solving – instruction order is more conducive to learning than the reverse order. Kapur and Bielaczyc (2012) have formulated three design principles for learning environments that promote the occurrence of productive failure effects: Firstly, an appropriate medium level of difficulty must be chosen for the learning tasks. It must be possible to draw on certain prior knowledge and to work on or solve the tasks in different ways. Secondly, the decisive features of the correct solution strategies must be explained and elaborated. Thirdly, learners should be enabled to compare the independently developed and incorrect solution strategies with the correct ones.

In deriving assumptions for learning, the productive failure approach, like the expertise-reversal effect, adopts a cognitive perspective. Kapur (2008) presumes a learning advantage of productive failure settings primarily in the preparatory character of the exploration phase, which makes better use of the instructional phase. He cites several explanations for this (Kapur, 2008, 2010, 2014). Firstly, relevant prior knowledge is identified and activated by the learner during the exploration phase (Kapur, 2010). This makes it more cognitively available in the subsequent instructional phase and the new information can be better classified and understood. Secondly, the learner can familiarize himself with the problem structure through the preliminary exercise, which makes orientation easier during the instruction phase (Kapur, 2010). Thirdly, the anticipated failure makes the learner aware of his knowledge gaps (Kapur, 2014). This enables the learner to direct his or her attention toward these gaps in the subsequent instructional phase. Fourth, in a productive failure condition, the learner is most likely to be confronted with his or her own erroneous solutions from the exploration phase which provides the opportunity to compare and contrast this kind of EE with CE presented in the consolidation phase (Kapur, 2014). This process can be very helpful in clarifying the crucial features of correct solution schemes. Fifth, the exploration phase trains the learner to be flexible in his problem-solving strategy in the long run when confronted with new problems and to continuously adapt the model to new information and circumstances (Kapur, 2008).

Several empirical studies have already been conducted to test these claims (e.g., Kapur, 2008, 2010, 2012, 2014; Kapur and Bielaczyc, 2012; Loibl and Rummel, 2014). All these studies showed significant learning differences as assumed by the productive failure approach. However, it must be critically noted that in these studies not only the order of the exploration and instruction phase but also other characteristics (may) varied between the conditions, and confounding is therefore possible (see also Ashman et al., 2020, for this reasoning). For example, one study, as a quasi-experiment, did not randomly assigned the experimental conditions but compared whole classes of students (Loibl and Rummel, 2014). In another study, group work was only facilitated in the productive failure condition (Kapur and Bielaczyc, 2012). Further studies used different teachers for carrying out the direct instruction phase or this phase was even carried out completely different in terms of content or format (Kapur, 2010, 2012; Kapur and Bielaczyc, 2012).

### Empirical findings on order effects

In the following, experimental studies that exclusively manipulated the order of more (e.g., explicit instruction or CE) and less guided (e.g., problem-solving) instructional methods will be discussed. The findings of these studies are quite inconsistent. Many findings support the expertise reversal effect as well as CLT and thus suggest that more guided instructional methods should be implemented first (Renkl et al., 2000, 2004; Atkinson et al., 2003; Renkl and Atkinson, 2003; Salden et al., 2010; Van Gog et al., 2011; Fyfe et al., 2014; Leppink et al., 2014; Hsu et al., 2015). For example, several studies investigated the guidance fading principle (Renkl et al., 2000, 2004; Atkinson et al., 2003; Renkl and Atkinson, 2003; Salden et al., 2010). In fading guidance conditions, learners receive only CE at the beginning of the learning phase, then they proceed with so-called completion tasks in which individual solution steps are missing, and at the end, they receive problem-solving tasks without any assistance. In all studies, this method proved to be more conducive to learning than a control group, which received multiple CE – problem pairs. Further studies indicate that CE should be presented before problem-solving tasks in the learning process rather than the other way around (Van Gog et al., 2011; Leppink et al., 2014). This order was found to lead to both better learning performance and lower mental effort. In addition, the study by Fyfe et al. (2014) indicates that conceptual instruction should be presented before problem-solving tasks as this order led to better problem-solving performance on a post-test.

However, there are also contradictory findings that support the productive failure approach and suggest that less guided methods should be implemented first (DeCaro and Rittle-Johnson, 2012; Kapur, 2014; Rittle‐Johnson et al., 2016; Lai et al., 2017; Weaver et al., 2018). Kapur (2014) investigated the effect of sequencing an instructional phase in which a teacher presented CE and problem-solving tasks on the topic of standard deviation and provided feedback on the latter and a pure problem-solving phase in which students were individually asked to collect as many solutions as possible. In the subsequent learning test, there were no differences between the groups in terms of problem-solving performance. However, students from the problem-solving – instruction condition performed better in terms of conceptual knowledge. Further studies confirm this finding concerning the expansion of conceptual knowledge (DeCaro and Rittle-Johnson, 2012; Lai et al., 2017; Weaver et al., 2018) but also partly concerning problem-solving performance (Lai et al., 2017), even though the two learning phases were sometimes presented differently in these studies. Sometimes, additional guiding aids were implemented in the problem-solving phase, such as the opportunity for collaborative exchange with classmates (Weaver et al., 2018) or corrective feedback (DeCaro and Rittle-Johnson, 2012). In another study, the direct instruction phase was made less guiding and more challenging by embedding several test questions (Lai et al., 2017).

Furthermore, several studies have been conducted examining the order of more and less guided methods in interaction with prior knowledge or topic complexity (Reisslein et al., 2006; Hsu et al., 2015; Ashman et al., 2020). Ashman et al. (2020) manipulated the sequence of explicit instruction and problem-solving. During instruction, a teacher explained to students how to calculate the energy efficiency of light bulbs. The students were also allowed to perform the calculations themselves and then participate in a discussion in which the teacher addressed, among other things, common erroneous solutions (comparable to EE). The first experiment showed that students from the explicit instruction – problem-solving order performed better on near transfer tasks in a delayed learning test a few days later. In a second experiment, the same design and learning topic was used again, but with more complex problem-solving. Here, the positive learning effect was confirmed with regard to the near transfer tasks and the same effect also occurred concerning the far transfer tasks. In the study by Hsu et al. (2015), a similar pattern emerged. They manipulated the order of CE and problem-solving and found an overall learning-promoting effect of the CE – problem-solving order. Subjects in this condition also reported lower mental effort. Furthermore, there was an interaction effect between order and expertise. The positive effect was only found for subjects who were classified as novices. In contrast, there was no difference for subjects who were classified as experts. Reisslein et al. (2006) found that expertise even reversed the direction of the order effect. Globally, there was no effect of order. However, subjects who were classified as novices benefited from the CE problem-solving order in a transfer test, in line with the previously discussed results. Subjects who were classified as experts, on the contrary, benefited from the problem-solving – CE order. These studies indicate that the contradictory findings on the effect of order described above may be explained by different levels of prior knowledge or element interactivity.

However, three research gaps emerge from the overall review of the literature on the order effects of more and less guided instructional methods. Firstly, while the order effects of explicit instruction and problem solving as well as of CE and problem-solving have been empirically investigated several times, there is no investigation of an order effect of CE and EE on learning performance and cognitive load. Secondly, apart from the variables of prior knowledge or element interactivity, no other moderating variables have yet been investigated that may change the influence of the order of more and less guided methods. Thirdly, no study has yet investigated whether the order of more and less guided instructional methods affects metacognitive variables such as the JOL. JOL refers to one’s self-assessment of the level of knowledge related to a particular learning topic and is thought to be a relevant factor in long-term learning success because it controls the future allocation of resources during self-regulated learning activities (Nelson and Dunlosky, 1991; Dunlosky and Hertzog, 1998; Zimmerman, 2008; Metcalfe, 2009). For example, the discrepancy-reduction model proposes that more learning resources are used for topics that are perceived as more difficult or for which a learner’s understanding is estimated to be lower (Dunlosky and Hertzog, 1998; Metcalfe, 2009). In this sense, JOL accuracy is considered decisive for self-regulated learning (Thiede et al., 2003; Zimmerman, 2008; Baars et al., 2017). If the JOL controls the distribution of resources, then it is most conducive if learner neither underestimate nor overestimate in their JOL, but correctly allocate the required resources for each subtopic (Thiede et al., 2003; Metcalfe, 2009). The present study aimed at these three research gaps described above.

### Hypotheses and research questions

Since CE can generally be classified as a guided instructional method and EE as a comparatively less guided instructional method, two theoretical perspectives were presented, which allow for the derivation of (contrasting) hypotheses regarding the question of whether more or less guided methods should be used first in the learning process. First of all, both, the expertise reversal effect and the productive failure approach assume that presenting less guided instructional methods first and more guided methods second exerts higher cognitive load than vice versa (Kalyuga, 2007; Kapur, 2014). This is empirically supported by studies that manipulated the order of explicit instruction and problem-solving tasks as well as those that manipulated the order of CE and problem-solving tasks (Van Gog et al., 2011; Kapur, 2014). Therefore, the following hypothesis was formulated:

*H1*: Learners in the CE-EE condition experience a lower cognitive load than learners in the EE-CE condition.

However, the two theoretical frameworks have contrasting suggestions for the effect of order on learning achievement. On the one hand, the expertise reversal effect (Kalyuga, 2007), following CLT (Sweller et al., 1998), claims that it is more conducive to learning if more guided instructional methods take place before less guided instructional methods. This assumption is supported by various findings (e.g., Van Gog et al., 2011). Moreover, studies show that expertise moderates the EE effect, and CE is more preferable when prior knowledge is low and EE is more preferable when prior knowledge is high (e.g., Große and Renkl, 2007). Therefore, according to the expertise reversal effect, it can be inferred that learners benefit from a CE-EE sequence. On the other hand, the productive failure approach (Kapur, 2008) predicts that it is more conducive to learning when less guided instructional methods precede more guided instructional methods. This assumption is also supported by several findings (e.g., Kapur, 2014). Therefore, according to the productive failure approach, it can be inferred that learners benefit from an EE-CE sequence. Due to the conflicting theoretical approaches and findings, the following research question was formulated:

RQ1: How does the order of CE and EE affect learning performance?

Furthermore, the two theories differ in their assumption about an indirect effect of order *via* cognitive load on learning achievement. On the one hand, the expertise reversal effect assumes that cognitive load caused by the design (e.g., order of methods) has a negative effect because the capacities of the working memory are strongly limited and thus can lead to an overload for some learners (Kalyuga, 2007). The productive failure approach, on the other hand, assumes that higher load after the entire learning phase should mainly be attributed to the high demands in the initial exploration and generation phase. Although this presents a difficulty for the learner, it can also be helpful because it supports schema assembly and helps the learner make better use of the subsequent instruction phase (Kapur, 2014). Due to the conflicting theoretical assumptions, following research question was formulated:

RQ2: Does cognitive load mediate the effect of the order of examples on learning performance?

Few studies examined the question of whether the influence of order is moderated by different variables (Reisslein et al., 2006; Hsu et al., 2015; Ashman et al., 2020). So far, these studies only identified prior knowledge and element interactivity as such variables. The present study aimed to investigate whether congruency between subsequent exemplified problems also interacts with the effect of order. According to cascade theory (VanLehn, 1999), EE are effective because they provoke impasses in which the learners are confronted with problems that they cannot solve immediately. If they first receive a CE and then an EE that exemplifies a different problem, they are likely to be prepared by the CE in finding and correcting the error and to do so more quickly. But the learners are still faced with an impasse as there is a problem that has to be solved first. However, if they first receive a CE and then an EE that exemplifies the very same problem the solution is already clear. Therefore, the learners will probably not deal with the EE in more depth. The beneficial reflection processes of EE according to the cascade theory are thus weakened. Therefore, the following hypothesis was formulated:

*H2*: The learning effectiveness of CE-EE order compared to EE-CE order is better if both examples exemplify different problems instead of the same problem.

As previously described, it has not yet been investigated whether the order of more and less guided instructional methods influences the metacognitive variables JOL and JOL accuracy, although these are very important for self-regulated learning. Social-cognitive accounts on self-regulated learning suggest that academic achievement under these circumstances is determined by the quality of self-observation, self-judgment, and self-reaction (Zimmerman, 1989; Bandura, 1991; Broadbent and Poon, 2015). Accordingly, people monitor their own performance and compare them with certain standards. Depending on the result, they form judgments about their performance and possibly regulate their learning activities in order to meet those standards. When working on different types of examples, learners should therefore observe how well they are understanding the exemplified procedures, thereby gathering information about their own abilities and forming a more or less accurate self-judgment. Moreover, the belief-adjustment model (Hogarth and Einhorn, 1992) proposes that the order in which different belief-relevant information is processed is crucial for belief formation. Depending on consistency, complexity, and length of information sets as well as response mode, primacy or recency effects can occur. JOL can be seen as one kind of belief formation as someone forms an opinion of one’s own state of learning. The learning experience with different instructional methods itself can be seen as relevant information for this belief formation. However, it is impossible to use the belief-adjustment model as a guiding framework for concrete hypotheses in the case of this study. One might determine *a priori* the length of the information set and the response mode but it is difficult to suggest if CE and EE are consistent in the information that they convey for JOL and to determine beforehand how complex the processing of these examples is. Furthermore, due to the lack of research, it is unclear which information CE and EE themselves convey for JOL and its accuracy. But since the model suggests that there might be any effect of order at all the following two research questions were formulated:

RQ3: How does the order of CE and EE influence JOL?

RQ4: How does the order of CE and EE influence JOL accuracy?

## Materials and methods

### Design and sample

The experiment followed a 2×2 between-subject design. The first factor represents the order (CE-EE vs. EE-CE) in which the CE and EE are presented. The second factor represents the congruence between the exemplified problems (same problems vs. different problems). Table 1 shows an overview of all experimental conditions. Before the study, an *a priori* power analysis was conducted using the software G*Power (Faul et al., 2007) for a 2 × 2 analysis of variance with a test power of 1-ß =0.80 and an alpha level of α = 0.05. Since no study has yet examined the effect of the order of CE and EE, the weighted average effect of the two studies that manipulated the order of CE and problem-solving tasks was taken as the estimated effect size: *f* = 0.34 (Van Gog et al., 2011; Leppink et al., 2014). The calculation resulted in a minimum sample size of *n* = 70.

**Table 1:** Descriptive statistics for all experimental conditions.

Variable	Unit	Experimental condition			
		Same problem		Different problems	
		CE-EE (*N =* 21)	EE-CE (*N =* 21)	CE-EE (*N =* 21)	EE-CE (*N =* 20)
		M (SD)	M (SD)	M (SD)	M (SD)
Learning (%)	1	52.38 (36.87)	58.57 (41.14)	61.67 (37.13)	46.50 (41.46)
	2	45.24 (40.08)	41.67 (34.98)	48.81 (37.08)	43.25 (29.92)
	3	42.14 (41.55)	28.81 (35.63)	32.14 (37.97)	51.50 (36.82)
Mental load	Overall	30.76 (7.99)	34.19 (6.00)	31.38 (5.30)	33.80 (5.43)
Mental effort	Overall	36.14 (5.84)	34.57 (6.71)	35.19 (4.57)	34.90 (6.50)
JOL_immediate_ (%)	1	21.95 (19.82)	31.10 (27.84)	26.29 (22.23)	30.35 (28.65)
	2	41.33 (30.20)	30.71 (29.41)	26.67 (27.36)	35.60 (27.28)
	3	27.33 (28.43)	24.00 (24.72)	13.71 (16.33)	19.60 (18.43)
JOL_delayed_ (%)	Overall	24.67 (21.93)	31.24 (26.85)	20.71 (13.72)	27.20 (22.98)
JOL accuracy_immediate_ (%)	1	−34.24 (26.72)	−30.81 (31.65)	−37.52 (35.68)	−25.65 (28.19)
	2	−4.86 (25.12)	−13.57 (23.96)	−22.86 (29.67)	−8.90 (38.33)
	3	−12.67 (28.16)	−5.52 (22.91)	−19.62 (34.73)	−34.40 (30.92)
JOL accuracy_delayed_ (%)	Overall	−21.05 (18.12)	−9.56 (17.88)	−25.48 (22.61)	−15.47 (24.24)

The maximum value for mental load and mental effort was 42. Overall means that the variable did not ask for a single unit but the whole learning session. CE, correct example; EE, erroneous example; JOL, judgment of learning.

The subjects were recruited *via* an e-mail distribution list of the Chemnitz University of Technology intended for study promotion. Current enrolment at a university was defined as a participation requirement to keep the sample as homogeneous as possible. Either eight euros or course credit was offered as compensation. A total of 86 students took part in the study. Two students were removed from the data set before the evaluation because they stated that they were not or no longer studying. Another student was removed because of technical difficulties during the survey that prevented the completion of one of the three learning units. The final sample size was therefore 83 students (72.0% female; age: *M* = 23.15; *SD* = 3.12). 89.15% of the students came from the [blinded for review]. 46.90% were enrolled in the Media Communication program, 29.62% in the Media and Instructional Psychology program, and 23.48% in other programs. Concerning the allowance, 69.88% opted for the course credit as compensation. Prior knowledge of the learning material of Vedic Mathematics was basically nonexistent. Only two participants stated that they had heard of the subject before, but were just like any other participant unable to solve any of the tasks in the subsequent pretest. Students were randomly assigned to one of the four conditions. There were no significant differences between the four conditions with regard to gender (*p* = 0.873), age (*p* = 0.649), university affiliation (*p* = 0.490), study program (*p* = 0.766), or prior knowledge (*p* = 0.585).

### Learning environment

The learning environment in this study consisted of a slideshow on Vedic Mathematics. Vedic Mathematics is an alternative calculation method invented by Tirthaji (1965), which can be used to solve rather complex mathematical problems relatively quickly without using a calculator. Three of these methods, also called rules, were selected as learning topics for this study and each formed an independent learning unit within the learning environment. The rule of the first unit was called “Vertical of powers of 10 and crosswise,” which concerned the multiplication of multi-digit numbers near powers of 10. The rule of the second unit was called “Vertical and crosswise,” which concerned the multiplication of any two-digit numbers. Lastly, the rule of the third unit was called “Division by 9 with remainder,” which concerned tasks where any number needs to be divided by nine.

The learning material contained a total of nine slides. Each unit contained three slides, one with direct instruction on the field of application and the methodology of the respective rule (see Appendix A), one slide with a CE, and one slide with an EE. The examples constituted the stimulus material. Which example was presented first and if the exemplified problems were the same or different was therefore dependent on the experimental condition. In addition, self-explanation prompts (“Try to understand why the examples are correct or incorrect and try to remember the correct solution steps for the test”) were inserted above the examples, as studies show that these are crucial for the learning effect of CE and EE (e.g., Chi et al., 1989; Große and Renkl, 2007; Hilbert and Renkl, 2009).

#### CE vs. EE

**Figure 1:**
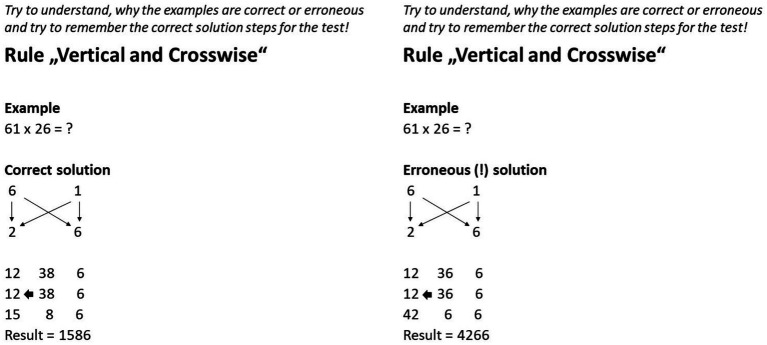
Slides with CE and EE in the *same* problem condition. The left example represents the CE and the right example represents the EE. Translated from German to English. Note that both examples were presented subsequently in the experiment.

**Figure 2:**
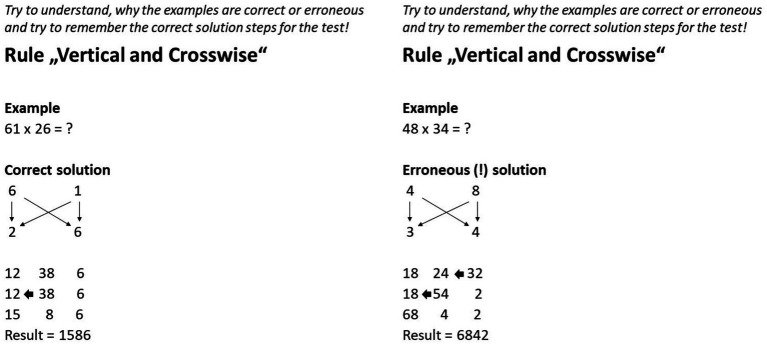
Slides with CE and EE in the *different* problems condition. The left example represents the CE and the right example represents the EE. Translated from German to English. Note that both examples were presented subsequently in the experiment.

Except for the subheadings that marked the solutions as correct or erroneous, the CE and EE differed only in the errors implemented (see Figures 1, 2). Since each of the three vedic rules can be roughly divided into two sub-steps, each EE contained two errors. Two criteria were applied in the selection of the errors. Firstly, to tie in with most other studies on EE, there should be conceptual errors instead of random calculation errors (e.g., Siegler, 2002; Große and Renkl, 2007). Secondly, the errors should not affect the comprehensibility of the rest of the solution steps so that the examples remained comparable apart from the errors. For example, this excluded errors such as the omission of a digit-rich calculation step, which might have led to the example becoming clearer overall. For example, the first error in the EE of the second learning unit was that the middle number only contained one crosswise product instead of the sum of both crosswise products (see Figure 1). The second error was that the tens were not correctly transferred from the right to the middle number and/or from the middle number to the left number as they were added to the respective tens instead of the respective ones (see Figure 1).

#### Same vs. different exemplified problems

The same and different problems conditions differed in terms of the correspondence between the two exemplified calculation problems presented. While in the same problems condition the numbers in the problems were identical and the CE thus represented the solution for the EE (see Figure 1), the numbers in the different problems condition differed (see Figure 2). Two different problems were therefore created for each learning unit. The numbers were so different under the two versions that the solution process was also slightly structurally different. As a consequence, it was not enough for the learner to simply replace numbers in the solution. When creating the different versions, further care was taken that the examples did not differ too much from each other in terms of difficulty, to avoid an unnecessary increment in variance within the groups. Since the difficulty of the selected calculation problems still could ultimately differ despite these considerations, the use of the two problems per unit was randomized within the four conditions to exclude this potentially confounding variable. For example, the two problems of the second unit “Vertical and Crosswise” were 61 × 26 =? and 48 × 34 =? The structural difference between the versions was that the former produced a one-digit number with the second vertical multiplication instead of a two-digit number as in the latter. This meant that, according to the vedic rule (see Figure 1), in the former only the tens of the middle sum had to be transferred while in the latter the tens of the right sum, as well as the tens of middle sum, had to be transferred.

### Measures

#### Cognitive load

To check the cognitive load, mental load and mental effort were recorded. The mental load (α = 0.80) and mental effort (α = 0.87) scales by Krell (2015) were used as a basis and adapted for the context of learning with examples (see Appendix B). Both scales consist of six items each, which must be rated on a seven-point Likert scale (1–do *not agree at all* and 7–*agree completely*). The mental load scale records the subjective assessment of the complexity and difficulty of the learning topic. For example, one item was “The examples were difficult to understand.” The mental effort scale, on the other hand, records the subjective assessment of the cognitive effort made by the learner. One item, for example, read “I have made an effort when working with the examples.”

The choice of scales considered recent conceptions of CLT (Sweller, 2010; Kalyuga, 2011; Sweller et al., 2019), which differentiate between several levels of analysis of cognitive processing: First, the analysis of cognitive load, which describes the theoretical demand of a learning environment and includes intrinsic load and extraneous load. On the other hand, the analysis of cognitive resources, which are actually used for processing and includes germane and extraneous processing. A differentiation of intrinsic load and extraneous load was not made, since the intrinsic load is independent of the learning design according to the CLT (Sweller et al., 1998) and thus any change in the extraneous load as a result of the manipulation should also be reflected in the level of the total load.

#### Learning achievement

For the assessment of learning achievement, a total of six problem-solving tasks were created, for each of which five points could be achieved. One point was awarded for each successfully completed step. Each unit was targeted by two problems, one problem_immediate_ which was presented immediately after the unit, and one problem_delayed_ which was presented delayed at the end of the experiment together with the two tasks which targeted the other units. The problems of the tasks always followed the problems of one of the respective exemplified problems of the unit. The internal consistency between all three problems_immediate_ (α = 0.32) and all three problems_delayed_ (α = 0.42) was very low. This can probably be explained by the different complexity of the learning units. On average, significantly different percent of the points were achieved for the test problems of the first learning unit, *M* = 54.88, *SD* = 54.88, of the second learning unit, *M* = 44.76, *SD =* 35.22, and of the third learning unit, *M* = 38.49, *SD* = 38.41 [*F*(2,82) = 4.93, *p* = 0.008, η_p_*^2^* = 0.06]. The both problems for each unit had good internal consistencies among themselves (*α* = 0.89; *α* = 0.88; *α* = 0.95). By respecting the internal consistency values and allowing a differentiated view of the effects with regard to the element interactivity, in the evaluation the learning achievement is considered separately by unit instead of point of presentation. For this purpose, the points from both corresponding problems were added together for each learning unit and divided by 10 (maximum number of points) to reach a percent value.

#### Metacognitive variables

A total of four scales were created to measure JOL. Three scales captured the JOL_immediate_ concerning one of each of the three learning units immediately after its completion: “What percentage of points do you think you would score in the following test item?.” The fourth scale recorded the JOL_delayed_ in relation to all three learning units after completion of the whole learning phase: “In your estimation, what percentage of points would you achieve in the following test on the application of the three vedic rules?”

JOL accuracy can be differentiated into relative and absolute JOL accuracy (Rhodes, 2016). In this study, absolute JOL accuracy was recorded because it indicates not only accuracy but also whether a learner underestimates or overestimates his or her own performance (Rhodes, 2016). Analog to JOL, four variables were calculated to measure JOL accuracy. Three variables represented the JOL accuracy_immediate_ concerning each of the three learning units. For this purpose, the percentage achieved in the immediate test task was subtracted from the corresponding JOL_immediate_. Positive values indicate overestimation and negative values underestimation. The fourth variable measured the JOL accuracy_delayed_ in relation to all three learning units after completion of the whole learning phase. For this purpose, the percentage achieved on the problems_delayed_ was subtracted from the JOL_delayed_. Positive values also indicate self-overestimation and negative values self-underestimation.

### Procedure

**Figure 3:**
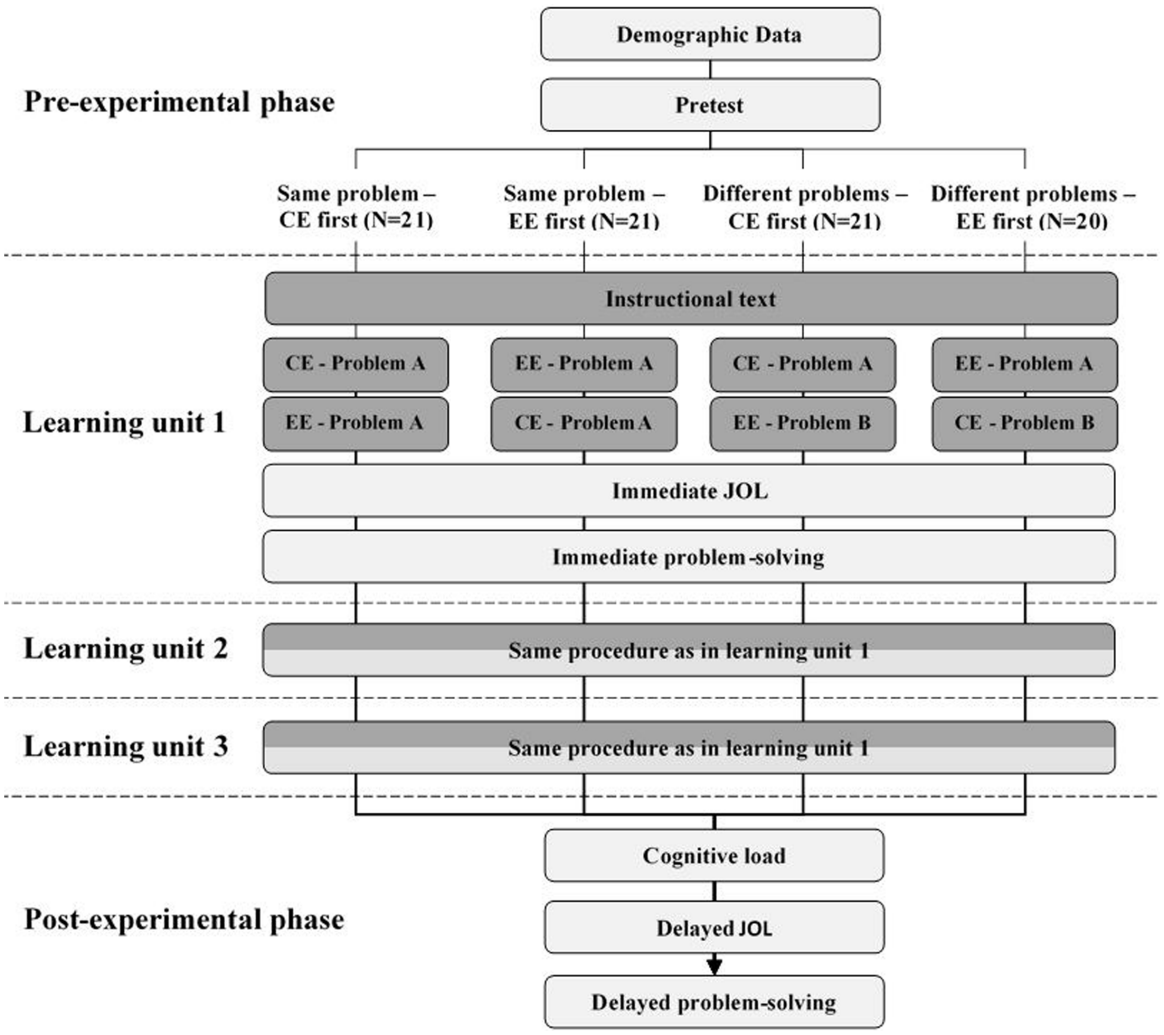
Flowchart for the four different experimental conditions. Dark boxes represent learning material, bright boxes represent measures. CE, correct example; EE, erroneous example; JOL, judgment of learning.

The study was conducted *via* an online conference system. After a short welcome and introduction, the participants were distributed into separate conference rooms and asked to share their browser windows with the investigator. This was to ensure that the subjects were exclusively engaged in the study and that no attempts at deception could take place. The participants accessed the learning environment *via* a link. Figure 3 illustrates the procedure with all important stages for all four conditions. After some demographic information, they were asked about their previous knowledge. On the next page, they were introduced in a few sentences to the topic of Vedic Mathematics and informed about the procedure of the study. Then the learning phase according to the experimental condition began. First, the unit “Vertical of powers of 10 and crosswise,” then the unit “Vertical and crosswise” and finally the unit “Division by 9 with remainder” were completed. Each unit contained a total of five pages. On the first three pages, the learning material was presented, first one page with explicit textual instruction and then two pages with one example each. If the CE or EE was presented first and also if the second example exemplified the same problem as the first example depending on the condition. For these three learning pages, the processing time was limited to 2 min per page. First, to ensure a high level of difficulty and variance in the results, and second, to avoid subjects spending so much time on a slide that they struggle to understand and consequently skip the rest of the slides. In the run-up to the study, the 2 min proved to be sufficient for independent testers to receive and elaborate on all the information on the slides at their leisure. Learners had the option to move on to the next page before the 2 min elapsed by pressing buttons labeled “Next” or “I have understood everything.” On the fourth page, the JOL_immediate_ for the respective rule was collected and on the last page, the problems_immediate_ for the respective rule was presented. After going through all three units, participants reported their mental load and mental effort. Then the JOL_delayed_ was collected. Finally, a block with the problems_delayed_ followed. After that, the experiment was finished.

## Results

Multivariate analyses of variance (MANOVA), univariate analyses of variance (ANOVA) as well as mediation analyses were conducted to test the hypotheses and research questions. If the MANOVAs produced at least marginally significant *F*-values, separate ANOVAs were then conducted to test the individual variables. In MANOVA and ANOVA, the experimentally manipulated variables order and congruency were included as factors. According to several authors (e.g., Glass et al., 1972; Ito, 1980), analyses of variance are robust to violations of the univariate and multivariate normal distribution and also robust to violations of variance homogeneity if the groups are similar in size, which is the case for this study. Therefore, we do not report these test requirements. Only in the case of MANOVA, violations of multicollinearity (*r* > 0.80; see Pituch and Stevens, 2019) would be reported, if existent. The descriptive statistics are presented for all four experimental conditions in Table 1. For mediational analysis (confidence interval = 95%, bootstrap resamples = 5,000), Preacher and Hayes (2008) indirect bootstrapping method was used. For all mediational models, order of examples was set as independent variable and either mental load or mental effort as mediating variable. Following recent analyses (e.g., Rucker et al., 2011; Loeys et al., 2015), a correlation between predictor and dependent variable, that means a significant total effect, was not considered as a prerequisite for testing indirect effects. To enhance clarity, single paths are not reported when the indirect effect was non-significant.

### Effects on cognitive load

A MANOVA with mental load and mental effort as dependent variables showed a marginally significant main effect for the factor order, Wilk’s Λ = 0.94, *F*(2,78) = 2.52, *p* = 0.087, η_p_^2^ = 0.06. However, there was neither a main effect for the factor congruence, Wilk’s Λ > 0.99, *F*(2,78) = 0.03, *p* = 0.968, η_p_^2^ < 0.01, nor an interaction effect, Wilk’s Λ > 0.99, *F* (2,78) = 0.19, *p* = 0.827, η_p_^2^ = 0.01. Separate ANOVAs were then conducted for the factor order. There was a significant medium effect on mental load, *F* (1,79) = 4.50, *p* = 0.037, η_p_^2^ = 0.05. The EE-CE group (*M* = 34.00, *SD =* 5.66, *N* = 41) reported higher mental load on average than the CE-EE group (*M* = 31.07, *SD =* 6.70, *N* = 42). There was no significant effect on mental effort, *F* (1,79) = 0.51, *p* = 0.479, η_p_^2^ = 0.01.

### Effects on learning achievement

A MANOVA with learning performance from the first, second, and third learning unit as dependent variables showed neither a main effect for the factor order, Wilk’s Λ = 0.99, *F* (3,77) = 0.29, *p* = 0.836, η_p_^2^ = 0.01, nor a main effect for the factor congruence, Wilk’s Λ = 0.99, *F*(3,77) = 0.21, *p* = 0.887, η_p_^2^ = 0.01. Furthermore, there was no interaction effect, Wilk’s Λ = 0.93, *F*(3,77) = 2.05, *p* = 0.117, η_p_^2^ = 0.07.

### Indirect effects *via* cognitive load on learning achievement

In total, six mediational analyses were conducted with two different mediators (mental load or mental effort) and three different dependent variables (problem_delayed_ for Learning unit 1, 2 or 3). Different from the other analyses, only the problem_delayed_ score was set as dependent variable to ensure antecedence in time of measurements since the immediate problem score was collected before the measurement of mental load and mental effort.

#### Mental load as mediator

For the first learning unit, the mediation analysis showed a significant partially standardized indirect effect of order *via* mental load on problem_delayed_, β = −0.22; *SE* = 0.11; 95% CI [−0.44, −0.03]. Overall, there was no significant total effect of order on problem_delayed_, β = −0.19; *p* = 0.398. Although, when mental load was added to the model the effect decreased, β = 0.03; *p* = 0.875. More specifically, presenting EE first and CE second resulted in higher mental load, β = 0.46; *p* = 0.035. Higher mental load in turn was associated with lower scores of problem_delayed_, β = −0.47; *p* < 0.001.

For the second learning unit, the mediation analysis showed a significant partially standardized indirect effect of order *via* mental load on problem_delayed_, β = −0.20; *SE* = 0.10; 95% CI [−0.39, −0.02]. Overall, there was no significant total effect of order on problem_delayed_, β = −0.15; *p* = 0.503. Although, when mental load was added to the model the effect decreased, β = 0.05; *p* = 0.819. More specifically, presenting EE first and CE second resulted in higher mental load, β = 0.46; *p* = 0.035. Higher mental load in turn was associated with lower scores of problem_delayed,_ β = −0.42; *p* < 0.001.

For the third learning unit, the mediation analysis showed a non-significant partially standardized indirect effect of order *via* mental load on problem_delayed_, β = −0.10; *SE* = 0.08; 95% CI [−0.29, 0.01].

**Figure 4:**
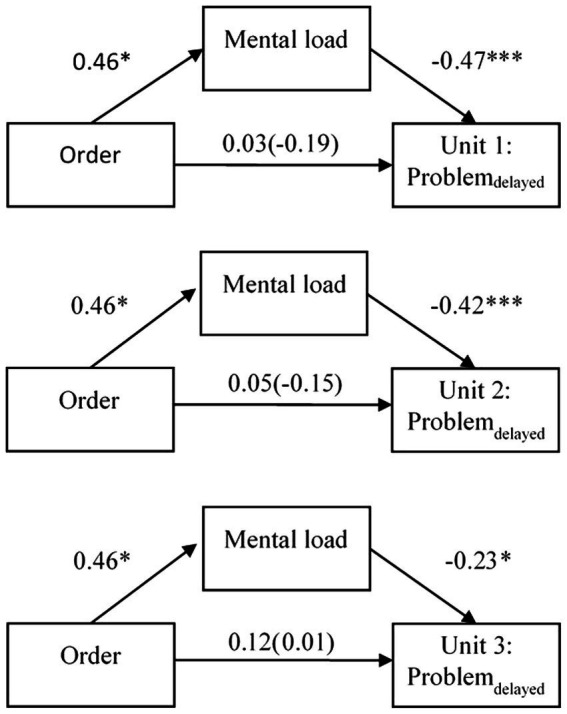
Path models with standardized β-coefficients and factor order of example types (correct example first vs. erroneous example first) as predictor, mental load as mediator, and problem_delayed_ as criterion for all three learning units. The total effect is reported in parentheses. *p < 0.05; ***p < 0.001.

See Figure 4 for graphical visualizations of all mediational models with mental load as mediator.

#### Mental effort as mediator

For the first learning unit, the mediation analysis showed a non-significant partially standardized indirect effect of order *via* mental effort on problem_delayed_, β = −0.03; *SE* = 0.05; 95% CI [−0.14, 0.07].

For the second learning unit, the mediation analysis showed a non-significant partially standardized indirect effect of order *via* mental effort on problem_delayed_, β = −0.03; *SE* = 0.05; 95% *CI* [−0.13, 0.07].

For the third learning unit, the mediation analysis showed a non-significant partially standardized indirect effect of order *via* mental effort on problem_delayed_, β = −0.04; *SE* = 0.06; 95% CI [−0.17, 0.09].

### Metacognitive variables

For JOL_immediate_, a MANOVA was conducted with the corresponding variables for the first, second, and third learning unit. There was no effect for the factor order, Wilk’s Λ = 0.98, *F*(3,77) = 0.54, *p* = 0.659, η_p_^2^ = 0.02. An ANOVA was performed for the JOL_delayed_. It showed no effect for the factor order, *F*(1,79) = 1.85, *p* = 0.178, η_p_^2^ = 0.02.

For JOL accuracy_immediate_, a MANOVA was conducted with the corresponding variables for the first, second, and third learning unit. There was no effect for the factor order, Wilk’s Λ = 0.98, *F*(3,77) = 0.59, *p* = 0.625, η_p_^2^ = 0.02. An ANOVA was conducted for the JOL accuracy_delayed_. There was a small, significant main effect for the factor order, *F*(1,79) = 5.52, *p* = 0.021, η_p_^2^ = 0.02. As indicated by confidence intervals, both the CE-EE (*M* = −23.26, *SD =* 20.37, *N =* 42, 95% CI [−29.67, −16,86]) and the EE-CE group (*M* = −12.44, *SD =* 21.16, *N* = 41, CI [−18.99, −6,03]) underestimated their performance on the problems_delayed_. However, the EE-CE group underestimated their performance to a lesser extent and their estimation was closer to the actual performance.

## Discussion

This study aimed to empirically test the opposing assumptions of the expertise reversal effect and the productive failure approach concerning the (indirect) effect of the order of CE and EE on learning performance. In addition, the aim was to investigate whether congruency between exemplified problems moderates the potential effect of order. Research questions and hypotheses on learning achievement, cognitive load (mental load, mental effort), and metacognitive assessment of learning performance (JOL and JOL accuracy) were formulated.

### Summary and implications

#### Findings on cognitive load and learning achievement

H1 could be partially supported. Contrary to the hypothesis, the order did not influence mental effort. However, subjects in the EE-CE conditions rated their mental load significantly higher than subjects in the CE-EE conditions. This result is consistent with studies that manipulated the order of other guided and less guided instructional methods (Van Gog et al., 2011; Kapur, 2014). It also confirms both the expertise reversal effect and the productive failure approach, both of which assume that the presentation of less guided measures at the beginning of the learning phase is associated with higher cognitive load (Kalyuga, 2007; Kapur, 2014; Ashman et al., 2020).

Concerning RQ1, the analysis showed no significant main effect of the order of the two types of examples on learning performance. However, concerning RQ2, the mediational analysis offered a more precise picture of the relationship between order of examples, cognitive load and learning as it indicated for two of three learning units that order may have no total or direct but an indirect effect *via* mental load on learning. In all three units, presenting EE first and CE second resulted in higher mental load. Additionally, higher mental load, in turn, was associated with lower scores on the problem-solving tasks for the first two learning units. As CE can be considered as a guided type of instruction and EE as a comparatively less guided type of instruction since at least one solution step has to be solved autonomously, this finding touches on the ongoing debate about the effectiveness of minimally guided and PS-I learning concepts (problem-solving first, instruction second) such as productive failure (e.g., Kirschner et al., 2006; Kapur, 2014; Loibl et al., 2017; Ashman et al., 2020). The non-significant total effect of order is in line with the so far inconsistent findings in this regard (e.g., Van Gog et al., 2011; Kapur, 2014). Nonetheless, the mediational analysis indicates that there are indeed pathways through which PS-I might have detrimental effects on learning, thereby supporting the CLT and the expertise reversal effect and challenging the idea of productive failure.

Contrary to mental load and the derivations from both, the expertise reversal effect and productive failure approach, mental effort neither acted as mediator nor was it significantly influenced by order. While both scales are used as indicators for cognitive load, mental load represents the perceived complexity of the learning material and mental effort represents the cognitive resources that have been actually devoted to learning the material. Therefore, it might be that the time limit during the learning phase was long enough to form an impression of difficulty but too short to offer variance in actual mental effort, thereby preventing significant effects.

H2 could not be confirmed. Congruency between examples had no moderating influence on learning performance. This result argues against cascade theory as an explanation for the learning effect of EE, which assumes that EE is effective in that it confronts learners with impasses where the correct solution step is not immediately recognizable, and thus reflection processes are being promoted (VanLehn, 1999). Following this explanatory approach, the positive effect would have been mitigated if, in a pair of examples that target the very same problem, the EE is presented after the CE (i.e., after the solution). However, this was not the case in this study and is possibly an indication that rather other mechanisms, such as the expansion of negative knowledge (knowing which solution strategies are wrong), are responsible for the learning-promoting effect of EE (Oser et al., 1999; Siegler, 2002; Große and Renkl, 2007).

For the practical design of learning environments, the findings suggest to present CE first and EE second in order to make the learning experience more cognitively pleasant and less exhausting for the learner. If EE have to be presented in early learning phases though, teachers should be cautious that the implemented errors are not too difficult to find and correct to prevent possible cognitive overload. When considering the actual learning achievement instead of learning experience though, the results of this study do not allow clear-cut recommendations for the order of CE and EE. On first glance, it seems that the order as well as the congruency of examples is not of great importance. Following this, the focus should be on making the examples themselves as conducive to instruct learners as possible. In addition, it could be useful to present the examples simultaneously and to ask the participants to compare them. This variant of presenting CE and EE has been shown in other studies to promote learning (Durkin and Rittle-Johnson, 2012; Heemsoth and Kleickmann, 2018; Loibl and Leuders, 2019). However, taking the mediational analysis into consideration, it seems that there is more to the effect of order on learning than the non-significant main effect would suggest. There might be ways in which order of examples does impact learning performance. Clearly more research is needed to further explore this relationship and to offer substantial recommendations for practitioners.

#### Findings on metacognitive variables

Concerning RQ3, order did not influence JOL. However, for RQ4, the EE first conditions were less likely to underestimate their delayed learning performance on all units than the CE first conditions. This effect of order may be interpreted in light of the belief-adjustment model (Hogarth and Einhorn, 1992). The model’s concrete assumptions depend on consistency, complexity, and length of information sets as well as response mode. *A priori* we could determine that as defined by Hogarth and Einhorn (1992) the length of information set was short (in total only six pieces of information). Additionally, we could classify the response mode as end-of-sequence mode since the JOL accuracy_delayed_ was collected only once after the entire learning phase ended. *A posteriori* we can now say, as indicated by the mental load ratings, that complexity of information was rather high. If we assume that CE and EE convey inconsistent information the model predicts a recency effect to bias the JOL accuracy, which means the last piece of information should have been given more weight in judgment formation. Since the EE-CE order condition was more accurate, it follows that CE might be better for estimating one’s own JOL. To the best of the author’s knowledge, so far there has been no theoretical or empirical work that focused on order effects of different instructional methods on JOL. Therefore, these results shed first light in this regard and have important practical implications. It follows for the practical design of learning environments that CE should succeed EE to improve the accuracy of self-assessment, especially if the presentation of examples concludes the lesson and self-regulated learning activities follow. In this case, it is vitally important for learners to correctly assess their current understanding of contents because this will guide them in allocating their study time for different subjects (Metcalfe, 2009). The trade-off with a higher cognitive burden for students during learning due to the CE-EE order might be considered individually.

The finding that the sample as a whole was quite bad in estimating their performance is in line with many other studies (Dunlosky and Lipko, 2007; Prinz et al., 2020). However, according to Dunlosky and Lipko (2007), learners usually overestimate rather than underestimate themselves, like in this study. This divergence could be explained by the fact that different from other studies, we set mathematical understanding instead of text comprehension as a learning goal (Dunlosky and Lipko, 2007; Prinz et al., 2020). In addition, the sample consisted largely of women. Consequently, some participants may have been biased through self-stereotyping when assessing their learning performance as bad mathematical ability is wrongly but commonly associated with the female gender (Watt, 2004; Burkley and Blanton, 2009). Interestingly, the overall underestimation of performance also contradicts research on the hard-easy effect which describes a well-documented bias in confidence ratings (Juslin et al., 2000; Moore and Healy, 2008). Although usually collected after a knowledge or performance test, confidence ratings are related to JOL as they represent the subjective confidence in or, differently termed, the judgment of one’s own specific knowledge. Supported by several empirical findings, the hard-easy effect proposes that difficult tasks foster overconfidence while easy tasks foster underconfidence (Juslin et al., 2000). As the mean performance on the problem-solving tasks was low to medium across all three units (see Table 1), the tasks difficulty can be considered as rather high. However, students still underestimated instead of overestimated their performance, contradicting the hard-easy effect. From this finding it follows that for predicting accuracy it might be decisive if one’s own knowledge base is evaluated before or after a test-situation.

### Limitations and future directions

The results of this study are limited in the sense that there are a few alternative explanations for the findings. Regarding the insignificant main effect of order, it can be argued from a productive failure perspective that not all conditions for the occurrence of an order effect were fully met. The productive failure effect has so far been found mainly in problem-solving tasks and direct instruction, but not in EE and CE. EE resembles problem-solving in that solutions have to be generated independently, but this usually applies only to a part and not to the whole task. Although, Kapur (2014) also conducted studies on vicarious failure in which learners, instead of generating solutions themselves, were presented with incorrect solutions by classmates and asked to evaluate them. This condition was found to be less beneficial than the classic productive failure problem-solving – direct instruction condition but still more beneficial than the direct instruction – problem-solving condition. Therefore, EE should at least in principle meet the criteria for a productive failure effect. Furthermore, unlike in the above-mentioned productive failure studies, we did not start with the exploratory method but with a short direct instruction phase. This was necessary so that the subjects had minimal prior knowledge of the topic and were able to recognize errors in the execution of the Vedic rules. However, this should not have prevented a true productive failure effect, since the EE themselves were followed by another form of guided instruction, namely the CE.

Secondly, the findings might only extend to learning environments that include EE with rather few errors. As the EE used in this study only include two errors the difference between EE and CE was rather small. This might have attenuated a possible effect and explain why no effect was found in contrast to former studies where the difference between the instructional methods used was more significant (e.g., Van Gog et al., 2011; Kapur, 2014). Future studies could include more errors in the EE to test this alternative explanatory approach.

Thirdly, it is possible that an effect of order did not occur because learners only focused on the CE when learning and tended to ignore the EE, for example, because the EE was judged to be too complex and difficult (Van Gog and Paas, 2008). This explanation is also supported by the finding that learners invested less time in learning with the EE (*M* = 59.87, *SD* = 35.52) than with the CE (*M* = 99.56, *SD* = 25.98), although EE should actually cause a higher cognitive load than CE and learners thus need more time to process them (McLaren et al., 2016). This finding also contradicts other studies that have compared the learning time of CE and EE (e.g., McLaren et al., 2016). In these studies, however, learners were also asked to correct the EE in writing. In this study, this was avoided to keep the conditions comparable. As a result, the implemented self-explanation prompt may have lost its effectiveness. Although collecting qualitative data was beyond the scope of this study, it might be recommendable for future studies to conduct post-hoc interviews with some participants and learn about their experience within the learning environment.

Regarding the significant indirect effects of order *via* mental load on learning, it must be carefully noted that a statistically significant mediation model does not ensure automatically that there actually is a causal relationship between the variables, as this assumption has to be met by the study’s method as well (Rohrer et al., 2022). In this study, however, only the predictor order had been manipulated experimentally, not the mediator mental load. Hence, the results might only be seen as an indication for a mediated model of effects. Future studies with a more suitable methodological framework are necessary to substantiate this finding. This could also help to shed light on the somewhat contradicting finding that there was no significant total effect of order on learning but a significant indirect effect *via* mental load.

In addition, the results of the study are limited concerning the interaction hypothesis between order and congruence. On the one hand, the null effect might be attributed to the low power of the study. The observed effect size was lower than expected and the *value of p* of the interaction almost reached marginal significance. Therefore, future studies with bigger sample sizes may find significant results. On the other hand, it can be assumed that there was no interaction effect because the learners did not have enough time to remember the several individual steps of the CE. As a result, and contrary to the original intention, the subjects in the congruent CE-EE condition might have failed to recognize the difference between the examples (i.e., the errors) early. This explanation is supported by the descriptive statistics of the learning units. In the first and simplest learning unit, where the steps could possibly be remembered more easily, the mean values of the groups tended to correspond to the postulated pattern. This was not the case in the other learning units. To test this alternative explanation, instead of two errors, only one error could be embedded in the EE in future studies. Or examples could be used that are clearer and do not require remembering so many calculation steps. Another possibility would be to use EE with concrete error signaling.

Moreover, as there has not been much theoretical and empirical work on the effect of order and congruency on metacognitive judgments, this study’s explanations for the significant findings on JOL accuracy need further elaboration. More data is needed to check on potential effects and their mechanisms. Using the belief-adjustment model (Hogarth and Einhorn, 1992) as a framework for explaining order effects on metacognitive judgments might be recommendable for future studies. Moreover, theoretical work is needed on the question of how CE and EE in isolation influence JOL and in case the order effect was indeed caused by recency bias why CE promote better JOL accuracy.

### Conclusion

This is the first study to experimentally investigate the effect of the order of correct and erroneous worked examples on learning performance as well as to examine whether congruency of exemplified problems functions as a moderator. Overall, the results show no total effect of order on learning but still indicate that there is an indirect effect. Presenting correct worked examples first and erroneous worked examples second resulted in lower mental load, which in turn was associated with better learning performance, thereby supporting the assumptions made by the expertise reversal effect and contradicting the assumptions made by the productive failure approach. Congruency did not moderate the impact of order on learning, challenging cascade theory as an explanation for erroneous worked example effects. Furthermore, order of examples significantly influenced JOL accuracy as learners estimated their learning performance more accurately when erroneous worked examples were presented first. Further studies are needed to test the limitations discussed above.

## Data availability statement

The raw data supporting the conclusions of this article will be made available by the authors, without undue reservation.

## Ethics statement

Ethical review and approval was not required for the study on human participants in accordance with the local legislation and institutional requirements. The patients/participants provided their written informed consent to participate in this study.

## Author contributions

LW designed the study, developed the materials, analyzed the data, and wrote the original manuscript. LW, FK, MB, and SS contributed to the research idea. FK, SJ, GR, MB, and SS revised the manuscript and critically improved its quality. SS supervised the entire process. All authors contributed to the article and approved the submitted version.

## Funding

The publication of this article was funded by Chemnitz University of Technology and Deutsche Forschungsgemeinschaft (DFG, German Research Foundation)–491193532.

## Conflict of interest

The authors declare that the research was conducted in the absence of any commercial or financial relationships that could be construed as a potential conflict of interest.

## Publisher’s note

All claims expressed in this article are solely those of the authors and do not necessarily represent those of their affiliated organizations, or those of the publisher, the editors and the reviewers. Any product that may be evaluated in this article, or claim that may be made by its manufacturer, is not guaranteed or endorsed by the publisher.

## Appendix A

### Explicit instruction

In the following you see the text which was provided on the slide with explicit instruction on the second learning unit “Vertical and Crosswise.”

### Application

Multiplication of two-digit numbers.

### Explanation

Both numbers are first written one below the other.

Then the respective first digits and the respective second digits are multiplied with each other. Afterwards the product of the first digit of the first number and the second digit of the second number is formed as well as the product of the second digit of the first number and the first digit of the second number. The sum of these two products is written between the products of the vertical multiplications.

Finally, the tens (if any) of the second vertical multiplication are transferred to the middle sum and the tens (if any) of this new number are transferred to the product of the first vertical multiplication. The result is made up of this new number and the two remainders.

## Appendix B

### Cognitive load survey

Adapted version according to Krell (2015). Translated from german to english.

### Mental load

- The examples were difficult to understand
- The content of the examples was complicated.
- The examples were challenging
- Working with the examples was easy
- The content of the examples was easy to understand.
- It was easy to understand why the examples were correct or incorrect.

### Mental effort

- When working with the examples I have not done my best particularly.
- I did not make a special effort to understand the examples.
- I have made an effort working with the examples.
- In understanding the examples, I made an effort intellectually.
- I have not particularly focused to understand why the examples were correct or incorrect.
- I have given my best to understand why the examples were correct or incorrect.
